# Acute kidney injury and renal replacement therapy: terminology standardization

**DOI:** 10.1590/2175-8239-JBN-2021-0284en

**Published:** 2022-05-18

**Authors:** Thiago Reis, Vinicius Sardão Colares, Eduardo Rocha, Mauricio Younes-Ibrahim, Emerson Quintino de Lima, Lucia da Conceição Andrade, Daniela Ponce, José H. Rocco Suassuna, Luis Yu

**Affiliations:** 1 Universidade de Brasília, Laboratório de Farmacologia Molecular, Brasília, DF, Brasil.; 2 Hospital DF Star, Clínica de Doenças Renais de Brasília, Brasília, DF, Brasil.; 3 Santa Casa de Misericórdia de Juiz de Fora, Juiz de Fora, MG, Brasil.; 4 Universidade Federal do Rio de Janeiro, Rio de Janeiro, RJ, Brasil.; 5 Universidade do Estado do Rio de Janeiro, Faculdade de Ciências Médicas, Rio de Janeiro, RJ, Brasil.; 6 Faculdade de Medicina de São José do Rio Preto, São José do Rio Preto, SP, Brasil.; 7 Universidade de São Paulo, Faculdade de Medicina, São Paulo, SP.; 8 Universidade Estadual de São Paulo, Departamento de Clínica Médica, São Paulo, SP, Brasil.

**Keywords:** Acute Kidney Injury, Renal Replacement Therapy, CKD, Chronic Kidney Disease, AKD, Acute Kidney Disease, Dialysis, Consensus, Injúria Renal Aguda, Terapia de Substituição Renal, DRC, Doença Renal Crônica, DRA, Doença Renal Aguda, Diálise, Consenso

## Abstract

The Department of Acute Kidney Injury (IRA) of the Brazilian Society of Nephrology prepared this document for the purpose of standardizing AKI terminology and dialysis modalities in the Portuguese language for Brazil. Several terms with similar meanings have been used in AKI and its dialysis modalities, causing confusion and disparities among patients, nephrologists, health institutions, private care companies, insurance companies and government entities. These disparities can impact medical care, hospital organization and care, as well as the funding and reimbursement of AKI-related procedures. Thus, consensual nomenclature and definitions were developed, including the definitions of AKI, acute kidney disease (AKD) and chronic kidney disease (CKD). Additionally, we addressed all dialysis modalities and extracorporeal procedures related to AKI, currently approved and available in the country. The Brazilian Society of Nephrology hopes that this Consensus can standardize the terminology and provide technical support to all involved in AKI care in Brazil.

## Resumo

O Departamento de Injúria Renal Aguda (IRA) da Sociedade Brasileira de Nefrologia elaborou o presente documento para fins de padronização da terminologia em IRA e modalidades dialíticas na língua portuguesa para o Brasil. Diversos termos com significados semelhantes têm sido empregados em IRA e suas modalidades dialíticas, causando confusão e disparidades entre pacientes, nefrologistas, instituições de saúde, empresas privadas de assistência, seguradoras e entidades governamentais. Essas disparidades podem impactar a assistência médica, a organização e o atendimento hospitalares, assim como o financiamento e reembolso dos procedimentos relacionados com a IRA. Assim, nomenclatura e definições consensuais foram elaboradas, incluindo-se as definições de IRA, doença renal aguda (DRA) e doença renal crônica (DRC). Adicionalmente, todas as modalidades dialíticas e os procedimentos extracorpóreos relacionados a IRA, atualmente aprovados e disponíveis no país, foram abordados. A Sociedade Brasileira de Nefrologia espera que este Consenso possa padronizar a nomenclatura e prover suporte técnico para todos os atores envolvidos na assistência à IRA no Brasil.

## Introduction

Acute kidney injury (AKI) is a frequent complication in hospitalized patients, especially in ICUs, still causing high rates of morbidity and mortality. AKI can also occur in outpatients and in the community, often related to socioeconomic and cultural conditions. Several terms with similar meanings have been used in AKI and its dialysis modalities, causing confusion and disparities among patients, nephrologists, healthcare institutions, private care companies, insurance companies and government entities. These disparities can impact medical care, hospital organization and care, as well as the funding and reimbursement of AKI-related procedures. Thus, we worked on the terminology and consensual definitions, including the definitions of AKI, acute kidney disease (AKD) and chronic kidney disease (CKD). Additionally, all dialysis modalities and extracorporeal procedures related to AKI, currently approved and available in the country, were addressed in this document by the AKI Department of the Brazilian Society of Nephrology.

## Acute Kidney Injury (AKI)

Preferably, use the term Acute Kidney Injury, in order to maintain the acronym AKI, which is well established and widespread in our country. In addition, the term was also approved in the Ibero-American Consensus on the Uniformity of Nomenclatures, observing the deliberations proposed by the Kidney Disease: Improving Global Outcomes (KDIGO) panel^1-8^. An additional advantage of using the term “injury” is that it encompasses initial cases with cellular and tissue functional alterations to cases of established anatomical lesions^9-16^. Therefore, it is suggested to avoid the term acute kidney lesion (AKL), which would be restricted to cases of anatomic kidney lesion.

We must also avoid the term “failure”, as it denotes a more advanced stage of renal failure.

## Kidney Failure (KF)

KF refers to a renal condition with a glomerular filtration rate lower than 15 mL/kg/1.73 m^2^ or when dialysis is required (Table 1)^3^.

**Table 1:** AKI and treatment terminology consensus

Suggested term	Suggested acronym	Justification	Terms to be avoided
Renal failure	RF	Glomerular filtration rate < 15 mL/kg/1,73 m^2^ or on dialysis	Terminal kidney disease; nephropathy; failure; azotemia
Duration			
Acute kidney injury stage 3	3D-stage AKI	Duration ≤ 3 months	Lesion/dysfunction/worsening/ renal failure; azotemia; uremia
Kidney insufficiency	KI	Duration > 3 months, evolving to chronic kidney disease (CKD)	Lesion/dysfunction/worsening/kidney failure; chronic nephropathy; azotemia; uremia; irreversible renal failure
Treatment			
Renal replacement therapy	RRT	Includes artificial renal support/dialysis and kidney transplant	
Artificial kidney support/dialysis	AKS	Stage 3- AKI under dialysis	AKI-D, dialysis-dependent AKI
Mode and frequency		Modes • hemodialysis (HD) • hemofiltration (HF) • hemodiafiltration (HDF) • peritoneal dialysis (manual or automated PD) Frequency • continuous • intermittent (conventional or Prolonged)	
Acute kidney disease (AKD) and acute kidney injury (AKI)		Illness lasting ≤ 3 months; differentiated from the chronic kidney disease (CKD)	Acute kidney lesion (AKL); acute kidney insufficiency; acute kidney failure
Acute kidney disease	AKD	KDIGO definition: AKI, or glomerular filtration rate < 60 mL/min/1.73 m^2^ or having kidney damage markers for a period ≤ 3 months, or glomerular filtration rate reduction ≥ 35% or serum creatinine increase > 50% during ≤ 3 months.	Acute kidney lesion (AKL); acute renal insufficiency; acute renal failure
Acute kidney injury	AKI	KDIGO definition (AKI is a subcategory of AKD): oliguria > 6 hours, serum creatinine increase > 0.3 mg/dL in 2 days or > 50% in one week.	Acute kidney lesion (AKL); acute renal insufficiency; acute renal failure
AKI classification		Classification by KDIGO stage and by etiology instead of by stage only; for instance, stage 3 AKI of septic/obstructive/hypotensive/hypovolemia etiology.	Old classifications with RIFLE and AKIN, once the KDIGO classification harmonized them.
AKI stages			
	Stage 1 AKI	Criteria: diuresis and/or serum creatinine	
	Stage 2 AKI	Criteria: diuresis and/or serum creatinine	
	Stage 3 AKI	Criteria: diuresis and/or serum creatinine	

## Renal Replacement Therapy (RRT)

RRT refers to any therapy associated with the process of replacing native kidney function, such as hemodialysis, peritoneal dialysis and kidney transplantation.

## Stage 3D Acute Kidney Injury

3D AKI is ARF requiring artificial renal support/dialysis. It is recommended to use the term 3D stage AKI instead of dialysis-dependent AKI.

## Artificial Kidney Support (AKS)

AKS encompasses all methods of artificial clearance.

Artificial renal support therapies are based on their modality and frequency:

### *Frequency*:

- **Continuous:** uninterrupted method of clearance, used with equipment with autonomy for uninterrupted operation of more than 24 hours.
- **Intermittent:** clearance method lasting less than 12 consecutive hours.

Intermittent therapies are subdivided into:

- **Conventional:** lasting up to 6 hours.
- **Prolonged:** lasting from 6 to 12 hours.

### *Modalities*:

The terminology in this text was based on the dialysis methods approved by ANVISA up to the date of document preparation.

## Extracorporeal artificial kidney support^3,17-22^

- Conventional hemodialysis.
- Conventional ultrafiltration.
- Conventional hemodiafiltration.
- Prolonged hemodialysis.
- Prolonged ultrafiltration.
- Prolonged hemodiafiltration.
- Continuous hemodialysis.
- Continuous hemofiltration.
- Continuous hemodiafiltration^23-26^.
- Continuous ultrafiltration^27-28^.

## Peritoneal artificial kidney support^29-32^

- Intermittent manual peritoneal dialysis.
- Continuous manual peritoneal dialysis.
- Intermittent automated peritoneal dialysis.
- Continuous low-volume automated peritoneal dialysis.
- High volume continuous automated peritoneal dialysis.

## Plasmapheresis^33-36^

- Therapeutic membrane plasmapheresis.
- Treatment plasmapheresis by centrifugation.

## Hemoperfusion^26,37-39^

- Hemoperfusion to remove medium molecules, drugs and toxins.
- Hemoperfusion to remove endotoxins.

## Artificial liver support^26,40,41^

- Recirculation system for molecular adsorption. Molecular adsorbent recirculating system (MARS).
- Single-pass albumin dialysis. Single-pass albumin dialysis (SPAD).
- Hemoperfusion to remove bilirubin and bile salts (uses the same system as hemoperfusion to remove medium molecules).

**Note**: Therapeutic plasma exchange may be considered as artificial liver support^33^.

## Removal of carbonic gas^26,42^

- Extracorporeal removal of carbon dioxide (CO_2_). Extracorporeal CO_2_ removal (ECCO_2_R).

## Extracorporeal artificial kidney support^3,17-22^

### Conventional hemodialysis

- Duration: up to 6 hours.
- Technique: hemodialysis.
- Removal mechanism: diffusion.
- Equipment: hemodialysis machine.
- Device: membrane filters with biocompatible polymer.
- Filter type: low flow, high flow, medium partition (medium cutoff) and high partition (high cutoff).
- Dialysis solution: polyelectrolytic concentrate for hemodialysis.
- Anticoagulation: heparin (unfractionated or low molecular weight), citrate or without anticoagulation.
- Place of treatment: preferably in individualized rooms, rooms for hemodialysis or in intensive and semi-intensive care units.

### Conventional ultrafiltration

- Duration: up to 6 hours.
- Technique: hemofiltration.
- Removal mechanism: convection.
- Equipment: hemodialysis machine and machines capable of performing ultrafiltration alone.
- Device: membrane filters with biocompatible polymer.
- Filter type: low flow, high flow, medium partition (medium cutoff) and high partition (high cutoff).
- Dialysis solution: not applicable.
- Anticoagulation: heparin (unfractionated or low molecular weight), citrate or without anticoagulation.
- Place of treatment: preferably in individualized rooms, rooms for hemodialysis or in intensive and semi-intensive care units.

### Conventional hemodiafiltration

- Duration: up to 6 hours.
- Technique: hemodialysis and hemofiltration.
- Removal mechanism: diffusion and convection.
- Equipment: hemodialysis machine with module for hemodiafiltration.
- Device: membrane filters with biocompatible polymer.
- Filter type: high flow.
- Dialysis and replacement solution: polyelectrolytic concentrate for hemodialysis.
- Anticoagulation: heparin (unfractionated or low molecular weight), citrate or without anticoagulation.
- Place of treatment: preferably in individualized rooms, hemodialysis rooms or in intensive and semi-intensive care units, with ultrapure water criteria.^41^

### Prolonged hemodialysis

- Duration: 6 to 12 hours.
- Technique: hemodialysis.
- Removal mechanism: diffusion.
- Equipment: hemodialysis machine.
- Device: membrane filters with biocompatible polymer.
- Filter type: low flow, high flow, medium partition (medium cutoff) and high partition (high cutoff).
- Dialysis solution: polyelectrolytic concentrate for hemodialysis.
- Anticoagulation: heparin (unfractionated or low molecular weight), citrate or without anticoagulation.
- Place of treatment: preferably in individualized rooms, rooms for hemodialysis or in intensive and semi-intensive care units.

### Prolonged ultrafiltration

- Duration: 6 to 12 hours.
- Technique: hemofiltration.
- Removal mechanism: convection.
- Equipment: hemodialysis machine and machines capable of performing ultrafiltration alone.
- Device: membrane filters with biocompatible polymer.
- Filter type: low flow, high flow, medium partition (medium cutoff) and high partition (high cutoff).
- Dialysis solution: not applicable.
- Anticoagulation: heparin (unfractionated or low molecular weight), citrate or without anticoagulation.
- Place of treatment: preferably in individualized rooms, rooms for hemodialysis or in intensive and semi-intensive care units.

### Prolonged hemodiafiltration

- Duration: 6 to 12 hours.
- Technique: hemodialysis and hemofiltration.
- Removal mechanism: diffusion and convection.
- Equipment: hemodialysis machine with module for hemodiafiltration.
- Device: membrane filters with biocompatible polymer.
- Filter type: high flow.
- Dialysis and replacement solution: polyelectrolytic concentrate for hemodialysis.
- Anticoagulation: heparin (unfractionated or low molecular weight), citrate or without anticoagulation.
- Place of treatment: preferably in individualized rooms, rooms for hemodialysis or in an intensive and semi-intensive care unit, with ultrapure water.

### Hemodialysis, hemofiltration and continuous hemodiafiltration^23-26^

- Duration: > 12 hours.
- Technique: hemodialysis, hemofiltration or hemodiafiltration.
- Removal mechanism: diffusion, convection and adsorption.
- Equipment: continuous hemodiafiltration machine with autonomy for uninterrupted operation for more than 24 hours.
- Device: membrane filters with biocompatible polymer.
- Filter type: high flow and high partition (high cutoff).
- Dialysis and replacement solution: specific electrolyte solutions for continuous hemodiafiltration.
- Anticoagulation: heparin (unfractionated or low molecular weight), citrate or without anticoagulation.
- Place of treatment: intensive and semi-intensive care units and surgical center.

### Continuous ultrafiltration^27,28^

- Duration: > 12 hours.
- Technique: hemofiltration.
- Removal mechanism: convection.
- Equipment: continuous hemodialysis machines and specific equipment for isolated ultrafiltration, with autonomy for uninterrupted operation of more than 24 hours.
- Device: membrane filters with biocompatible polymer.
- Filter type: low or high flow.
- Dialysis and replacement solution: not applicable.
- Anticoagulation: heparin (unfractionated or low molecular weight), or without anticoagulation.
- Place of treatment: intensive and semi-intensive care units and surgical center.

## Peritoneal artificial kidney support^29-32^

### Intermittent manual peritoneal dialysis

- Duration: up to 12 hours.
- Volume: up to 12 L.
- Technique: peritoneal dialysis.
- Removal mechanism: diffusion.
- Equipment: not applicable.
- Solutions: hypertonic glucose or icodextrin.
- Anticoagulation: not applicable.
- Place of treatment: preferably in individualized rooms, rooms for hemodialysis or in intensive and semi-intensive care units.

### Continuous manual peritoneal dialysis

- Duration: > 12 hours.
- Standard volume: up to 12 L.
- Technique: peritoneal dialysis.
- Removal mechanism: diffusion.
- Equipment: not applicable.
- Anticoagulation: not applicable.
- Solutions: hypertonic glucose, icodextrin.
- Place of treatment: preferably in individualized rooms, rooms for hemodialysis or in intensive and semi-intensive care units.

### Intermittent automated peritoneal dialysis

- Duration: up to 12 hours.
- Standard volume: up to 12 L.
- Technique: peritoneal dialysis.
- Removal mechanism: diffusion.
- Equipment: cycling machine.
- Solutions: hypertonic glucose or icodextrin.
- Anticoagulation: not applicable.
- Place of treatment: preferably in individualized rooms, rooms for hemodialysis or in intensive and semi-intensive care units.

**Note:** the term “automated” implies the need for a specific machine for this therapy.

### Low volume continuous automated peritoneal dialysis

- Duration: > 12 hours.
- Standard volume: up to 12 L.
- Technique: peritoneal dialysis.
- Removal mechanism: diffusion.
- Equipment: cycling machine.
- Solutions: hypertonic glucose.
- Anticoagulation: not applicable.
- Place of treatment: preferably in individualized rooms, rooms for hemodialysis or in intensive and semi-intensive care units.

### High volume continuous automated peritoneal dialysis

- Duration: > 12 hours.
- Standard volume: > 12 L up to 42 L.
- Technique: peritoneal dialysis.
- Removal mechanism: diffusion.
- Equipment: cycling machine.
- Solutions: hypertonic glucose.
- Anticoagulation: not applicable.
- Place of treatment: preferably in individualized rooms, rooms for hemodialysis or in intensive and semi-intensive care units.

## Plasmaferesis^33-36^

### Treatment membrane plasmaferesis

- Duration: 2-6 hours.
- Technique: hemofiltration.
- Removal mechanism: convection limited by the size of the molecule.
- Equipment: hemodialysis or continuous hemodiafiltration machine with plasmapheresis module.
- Device: membrane filters with biocompatible polymer.
- Filter type: plasma filter.
- Replacement solution: solution with human albumin, fresh frozen plasma or cryoprecipitate.
- Anticoagulation: heparin (unfractionated or low molecular weight), citrate or without anticoagulation.
- Place of treatment: preferably in individualized rooms, rooms for hemodialysis or in intensive and semi-intensive care units.

### Centrifuge plasmapheresis

- Duration: 2-6 hours.
- Technique: centrifugation.
- Removal mechanism: sedimentation by specific gravity.
- Equipment: centrifuge machine.
- Device: not applicable.
- Filter type: not applicable.
- Replacement solution: solution with human albumin, fresh frozen plasma or cryoprecipitate.
- Anticoagulation: heparin (unfractionated or low molecular weight), citrate or without anticoagulation.
- Place of treatment: preferably in individualized rooms, rooms for hemodialysis or in intensive and semi-intensive care units.

## Hemoperfusion^26,37-39^

### Hemoperfusion for removal of medium molecules (0.5 - 58 Kda), drugs and toxins

- Duration: 2-24 hours.
- Technique: hemoperfusion.
- Removal mechanism: adsorption.
- Equipment: hemodialysis machines, continuous procedure machines or specific machines for hemoperfusion, or cartridges connected to the extracorporeal circulation circuit or to the extracorporeal membrane oxygenation (ECMO) circuit, in these situations without the need for specific equipment.
- Device: cartridges with resins or microspheres.
- Filter type: not applicable.
- Dialysis and replacement solution:
- In case of use of a specific machine for hemoperfusion: not applicable.
- If using a hemodialysis or hemofiltration machine: specific electrolyte solutions.
- In case of use of continuous hemodiafiltration machine: specific electrolyte solutions for continuous hemodiafiltration.
- Anticoagulation: heparin (unfractionated or low molecular weight), citrate or without anticoagulation.
- Place of treatment: preferably in individualized rooms, rooms for hemodialysis, intensive care unit, semi-intensive care unit and surgical center.

### Hemoperfusion for endotoxin removal

- Duration: 2-4 hours.
- Technique: hemoperfusion.
- Removal mechanism: adsorption.
- Equipment: specific machine for hemoperfusion, hemodialysis machine or continuous hemodiafiltration connected in series with the pre- or post-filter extracorporeal circuit.
- Device: cartridge with synthetic resins with specific adsorptive capacity.
- Filter type: not applicable.
- Dialysis and replacement solution:
- In case of use of a specific machine for hemoperfusion: not applicable.
- If using a hemodialysis or hemofiltration machine: specific electrolyte solutions.
- In case of use of continuous hemodiafiltration machine: specific electrolyte solutions for continuous hemodiafiltration.
- Anticoagulation: heparin (unfractionated or low molecular weight), citrate or without anticoagulation.
- Place of treatment: intensive and semi-intensive care unit.

## Artificial hepatic support^26,40,41^

### Molecular adsorption recirculation system (mars)

- Duration: 6-8 hours.
- Technique: hemodialysis and perfusion for regeneration of the albumin solution.
- Removal mechanism: diffusion and adsorption by albumin.
- Equipment: specific machine coupled to a continuous hemodiafiltration or hemodialysis machine.
- Device: membrane filters with biocompatible polymer, with parallel circuit of activated carbon cartridge and ion exchanger cartridge, for recirculation of solution with albumin.
- Type of filter: low flow (for albumin regeneration) and high flow (for the passage of blood inside the capillary fibers in countercurrent with the albumin solution).
- Dialysis and replacement solution: specific electrolyte solutions for continuous hemodiafiltration. Human albumin solution for the parallel circuit.
- Anticoagulation: heparin (unfractionated or low molecular weight), citrate or without anticoagulation.
- Place of treatment: intensive and semi-intensive care unit.

### Single pass albumin dialysis (spad)

- Duration: 6-8 hours.
- Technique: hemodialysis.
- Removal mechanism: diffusion.
- Equipment: continuous hemodiafiltration machine with autonomy for uninterrupted operation for more than 24 hours.
- Device: membrane filters with biocompatible polymer.
- Filter type: high flow and high partition (high cutoff).
- Dialysis solution: specific electrolyte solutions for continuous hemodiafiltration with the addition of human albumin.
- Anticoagulation: heparin (unfractionated or low molecular weight), citrate or without anticoagulation.
- Place of treatment: preferably in individualized rooms, rooms for hemodialysis or in intensive and semi-intensive care units.

### Hemoperfusion for bilirubin and biliary salts removal

- Duration: 2-24 hours.
- Technique: hemoperfusion.
- Removal mechanism: adsorption.
- Equipment: hemodialysis machines, continuous procedure machines or specific machines for hemoperfusion, or cartridges connected to the extracorporeal circulation circuit or to the extracorporeal blood oxygenation (ECMO) circuit, in these situations without the need for specific equipment.
- Device: cartridges with resins or microspheres.
- Filter type: not applicable.
- Dialysis and replacement solution:
- In case of use of a specific machine for hemoperfusion: not applicable.
- If using a hemodialysis or hemofiltration machine: specific electrolyte solutions.
- In case of continuous hemodiafiltration machine use: specific electrolyte solutions for continuous hemodiafiltration.
- Anticoagulation: heparin (unfractionated or low molecular weight), citrate or without anticoagulation.
- Place of treatment: preferably in individualized rooms, rooms for hemodialysis, intensive care unit, semi-intensive care unit and surgical center.

## Removal of carbonic gas^26,42^

### Extracorporeal carbon dioxide removal (ECCO_2_R)

- Duration: >48 h.
- Technique: hemodialysis.
- Removal mechanism: diffusion of gases.
- Equipment: specific machine for hemoperfusion, continuous hemodiafiltration machine coupled to the oxygenating membrane.
- Device: oxygenating membrane.
- Filter type: not applicable.
- Sweep gas: oxygen.
- Dialysis and replacement solution:
- In case of use of a specific machine for hemoperfusion: not applicable.
- In case of use of continuous hemodiafiltration machine: specific electrolyte solutions for continuous hemodiafiltration.
- Anticoagulation: heparin (unfractionated or low molecular weight) or without anticoagulation.
- Place of treatment: intensive care unit.

## Conclusions

The AKI Department of the Brazilian Society of Nephrology prepared this document in order to standardize the terminology and definitions related to AKI.

Terminology and consensual definitions were addressed, including definitions of AKI, acute kidney disease (AKI) and chronic kidney disease (CKD). All dialysis modalities and extracorporeal procedures related to AKI, currently approved and available in the country, were described. The Brazilian Society of Nephrology hopes that this Consensus can standardize the terminology and provide technical support to all sectors involved in AKI assistance in Brazil.
